# Oxidative stress mediates depot-specific functional differences of human adipose-derived stem cells

**DOI:** 10.1186/s13287-019-1240-y

**Published:** 2019-05-21

**Authors:** Sandhya Sriram, Chengxiang Yuan, Smarajit Chakraborty, Winson Tay, Min Park, Asim Shabbir, Sue-Anne Toh, Weiping Han, Shigeki Sugii

**Affiliations:** 1 Fat Metabolism and Stem Cell Group, Singapore Bioimaging Consortium (SBIC), Agency for Science, Technology and Research (A*STAR), 11 Biopolis Way #02-02, Singapore, 138667 Singapore; 2 Duke-NUS Medical School, 8 College Road, Singapore, 169857 Singapore; 3 Department of Surgery, National University Hospital, 5 Lower Kent Ridge Road, Singapore, 119074 Singapore; 4 Department of Medicine, Yong Loo Lin School of Medicine, National University of Singapore, 14 Medical Drive, Singapore, 117599 Singapore; 5 Laboratory of Metabolic Medicine, Singapore Bioimaging Consortium (SBIC), Agency for Science, Technology and Research (A*STAR), 11 Biopolis Way, Singapore, 138667 Singapore; 6 Present address: Institute of Bioengineering and Nanotechnology (IBN), Agency for Science, Technology and Research (A*STAR), 31 Biopolis Way #07-01, Singapore, 138669 Singapore

**Keywords:** Subcutaneous fat, Intra-abdominal fat, Mesenchymal stromal cells, Oxidative stress, Reactive oxygen species, Ascorbic acid

## Abstract

**Background:**

Visceral (VS) fat depot is known to have defective adipogenic functions compared to subcutaneous (SC) fat, but its mechanism of origin is unclear.

**Objective:**

We tested our hypothesis that the degree of oxidative stress in adipose-derived stem cells (ASCs) from these depots may account for this difference.

**Methods:**

ASCs were isolated from VS (omental region) and SC (abdominal region) fat depots of human subjects undergoing bariatric surgery. ASCs from VS and SC fat were investigated for their cellular characteristics in reactive oxygen species (ROS), metabolism, gene expression, proliferation, senescence, migration, and adipocyte differentiation. ASCs were also treated with antioxidant ascorbic acid (vitamin C).

**Results:**

We found that human VS-derived ASCs exhibit excessive oxidative stress characterized by high reactive oxygen species (ROS), compared to SC-derived ASCs. Gene expression analyses indicate that the VS-ASCs exhibit higher levels of genes involved in pro-oxidant and pro-inflammatory pathways and lower levels of genes in antioxidant and anti-inflammatory pathways. VS-ASCs have impaired cellular functions compared to SC-ASCs, such as slower proliferation, early senescence, less migratory activity, and poor adipogenic capability in vitro. Treatment with ascorbic acid decreased ROS levels drastically in VS-ASCs. Ascorbic acid treatment substantially improved proliferation, senescence, migration, and adipogenic capacities of compromised ASCs caused by high ROS.

**Conclusions:**

This finding suggests the fat depot-specific differences of cellular defects originating from stem cell population. Considering clinical potentials of human ASCs for cell therapies, this also offers a possible strategy for improving their therapeutic qualities through antioxidants.

**Electronic supplementary material:**

The online version of this article (10.1186/s13287-019-1240-y) contains supplementary material, which is available to authorized users.

## Introduction

Adipose-derived stem cells (ASCs) are the mesenchymal stem cell (MSC) type that exhibits cellular characteristics potentially useful for regenerative medicine, which includes multipotent and migratory capacities [1, 2]. Due to the relative ease in isolation, abundance, and therapeutic potencies, a number of clinical trials involving ASCs are currently undertaken worldwide in diverse disease indications [3]. Within white fat tissue, ASCs are prevalent and believed to be the origin of mature adipocytes [1, 4]. There are at least two different types of white adipose tissue, subcutaneous (SC) fat depot residing under the skin and visceral (VS) fat depot surrounding internal organs, which exhibit distinct adipogenic phenotypes. For example, in contrast to SC fat, VS fat is characterized by poor lipid storage capacity, higher inflammation, altered adipokine release, and insulin resistance [5, 6]. As a result, the expansion of VS adiposity during obesity is associated with various metabolic complications including diabetes, hypertension, hyperlipidemia, and cardiovascular diseases. It was previously demonstrated in rodents and human that progenitor and stem cell population show fat depot-specific differences in adipogenic capacities in vitro [7–10]. Stem cells from VS fat are poorly differentiated into mature adipocytes compared to SC-derived cells, but molecular mechanisms underlying this difference are incompletely understood. Oxidative stress resulting from increased reactive oxygen species (ROS) is known to cause cellular damages leading to genomic instability, senescence, and apoptosis. The involvement of oxidative stress in obesity and diabetes is relatively well studied; numerous studies have been performed on diet-induced obesity animal models and human obese patients to determine the effects of oxidative stress/ROS levels in metabolic organs and systemic metabolism [11–15]. Functions of fat tissue and adipocytes are greatly affected by intracellular ROS levels, though mechanisms leading to the oxidative stress in fat are still not clear [16]. Little is known about the ROS profiles and its effect on cells derived from the anatomically distinct SC and VS fat depots. In this manuscript, we investigated possible roles of oxidative stress in mediating depot-specific differences in human ASCs.

## Results and discussion

**Fig. 1:**
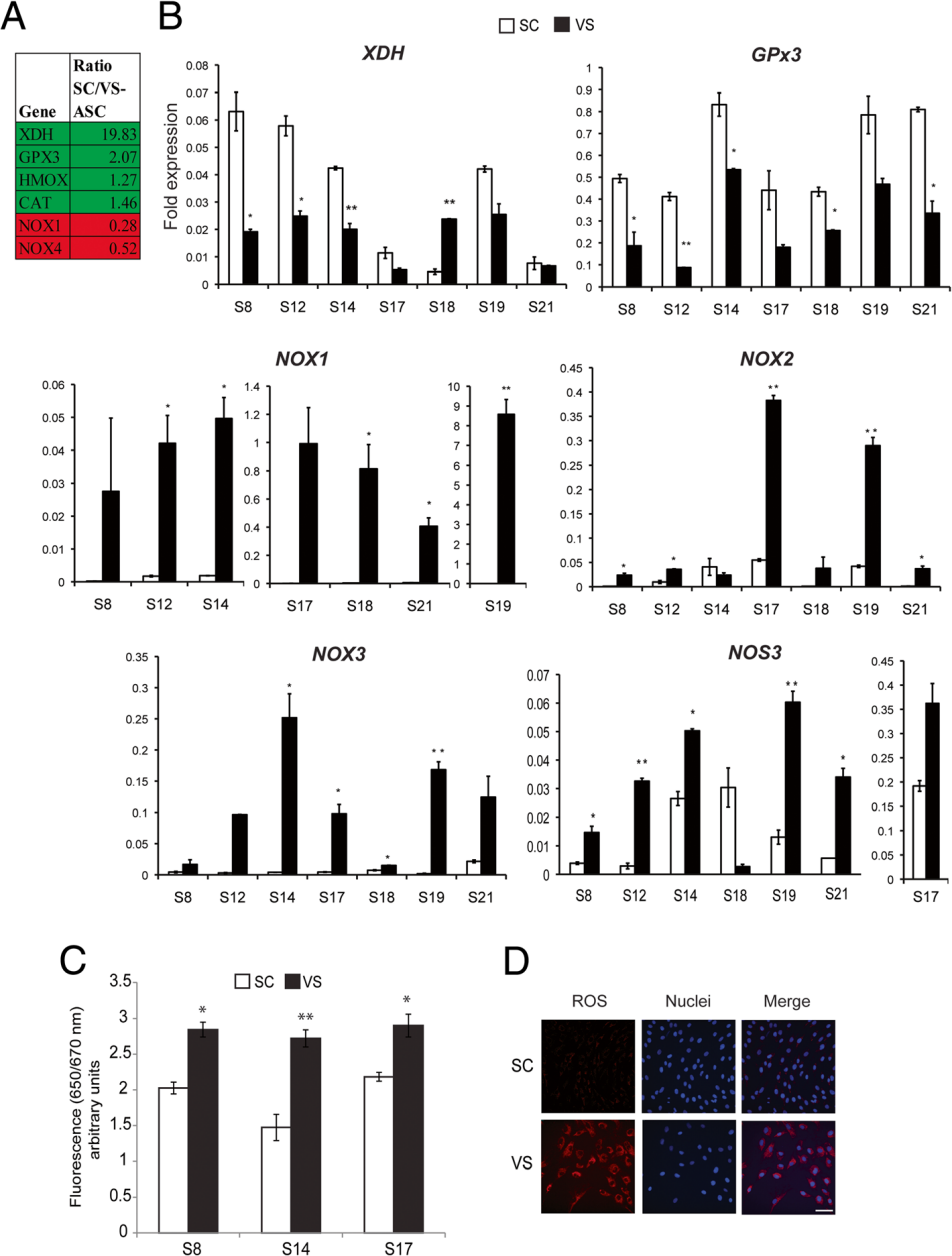
VS-ASCs have increased ROS when compared to SC-ASCs. **a** Table showing fold expression of genes differentially expressed in SC- vs. VS-ASCs from the microarray data. **b** Graphs showing fold expression of oxidative stress-related genes *XDH*, *GPx3*, *NOX1*, *NOX2*, *NOX3*, and *NOS3* each in triplicates in SC- and VS-ASCs from seven subjects. Similar results were obtained from other subjects. Statistical significance was calculated by ANOVA. *p < 0.05, **p < 0.01, and ***p < 0.001 when compared to SC. **c** Graph showing fluorescence intensity of SC- and VS-ASCs (p8) each in triplicates from three representative subjects when stained with CellROX™ Deep Red Reagent. Statistical significance was calculated by ANOVA. *p < 0.05 and **p < 0.01 when compared to SC. **d** Representative images (× 40) showing fluorescence staining of ROS (red) and nuclei (blue) in S17 SC- and VS-ASCs when stained with CellROX™ and Hoechst 33342, respectively. Scale bar represents 100 μm

In our previous publication [10], we performed microarray and ontological analysis on human ASCs isolated from SC and VS depots, in order to identify genes underlying their molecular differences. We recognized a set of genes involved in either generating or detoxifying ROS to be differentially expressed in SC- and VS-ASCs, as shown in Fig. 1a. We performed quantitative real-time PCR analysis and confirmed that a subset of those genes are consistently and differentially expressed across different subjects (Fig. 1b). SC-ASCs show upregulation of genes implicated in antioxidant activities including xanthine dehydrogenase (*XDH*) and glutathione peroxidase 3 (*GPx3*) in the majority of subjects. On the other hand, VS-ASCs generally have upregulated expression of oxidative stress-inducing genes such as NADPH oxidases 1, 2, and 3 (*NOX1*, *NOX2*, *NOX3*) and nitric oxide synthase 3 (*NOS3*). Among those found in microarray analysis, antioxidants catalase (*CAT*) and heme oxygenase-1 (*HMOX1*) were higher in five out of seven SC-ASCs (Additional file 1: Figure S1). Other oxidative stress-related genes, such as *NOX4*, *NOX5*, *GPx1*, *GPx2*, *GPx4*, *SOD1*, *SOD2*, and *SOD3*, exhibit variable expressions across subjects, indicating that different factors may influence these genes to varying degrees (Additional file 1: Figure S1). Based on expression of these genes, we anticipated higher oxidative stress in VS-ASCs. By using a specific fluorescent dye, we confirmed that reactive oxygen species (ROS) is present at significantly higher levels in VS-ASCs than in SC-ASCs (Fig. 1c, d). The same finding was observed when low passage VS-ASCs and SC-ASCs were compared (Additional file 1: Figure S2).

**Fig. 2:**
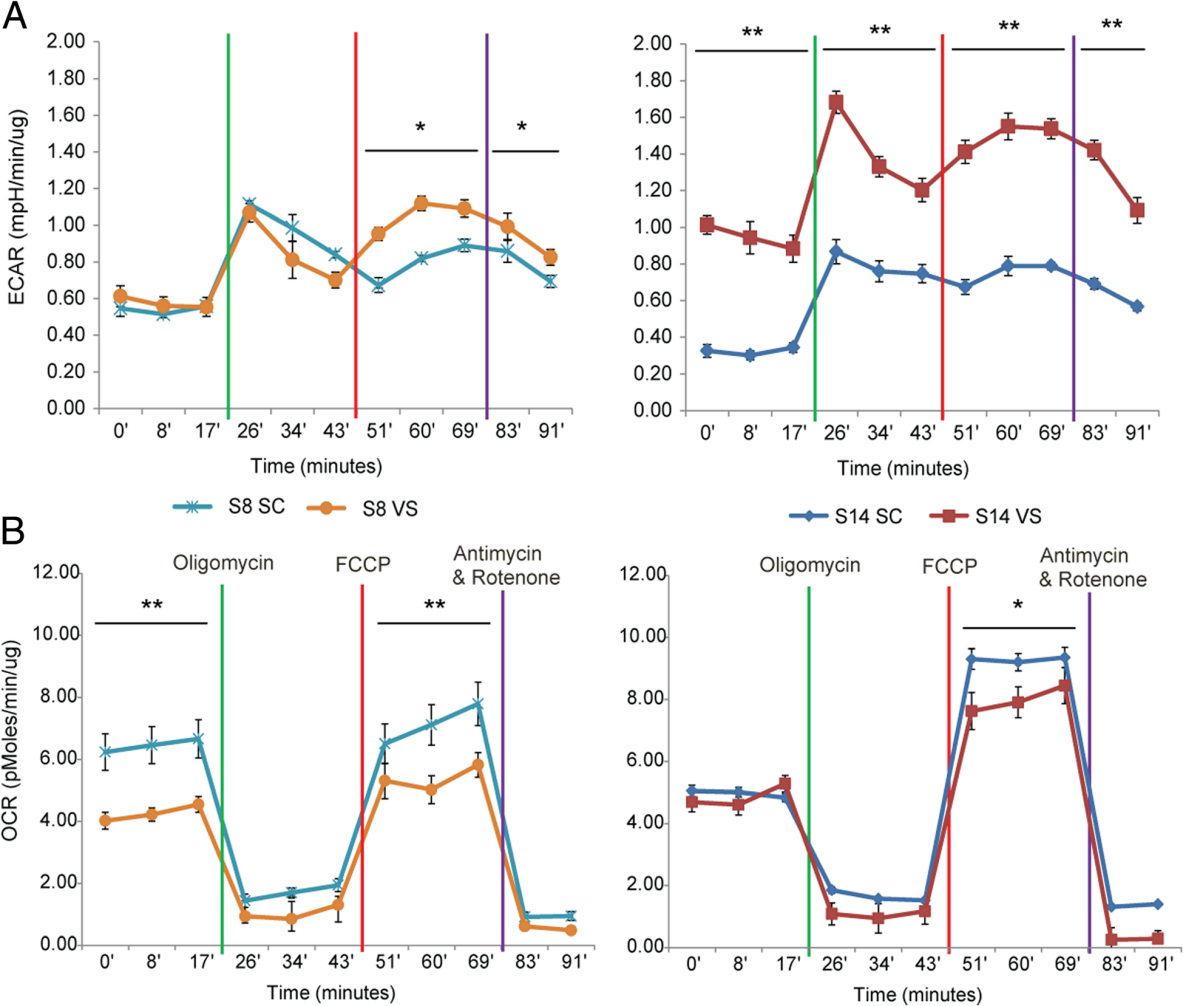
ASCs exhibit depot-specific differences in mitochondrial respiration and glycolysis. **a** Graph showing ECAR (extracellular acidification rate) by Seahorse XF analyses each in triplicates for SC- and VS-ASCs from two representative subjects (S8 and S14). **b** Graph showing OCR (oxygen consumption rate) by Seahorse XF analyses each in triplicates for SC- and VS-ASCs from two subjects. Statistical significance was calculated by ANOVA. *p < 0.05, **p < 0.01

Seahorse XF Analyzer was employed to investigate glycolytic and oxidative metabolism of SC- and VS-ASCs. As shown in Fig. 2, the extracellular acidification rate (ECAR) tends to be increased in VS-ASCs compared to SC-ASCs, suggesting that VS-ASCs generally have higher glycolysis activity (Fig. 2a). In contrast, VS-ASCs show a lower oxygen consumption rate (OCR) than SC-ASCs, especially under maximal respiratory states, indicating that VS-ASCs have less oxidative metabolism capacity (Fig. 2b). Increased ROS leads to mitochondrial dysfunctions, which is observed in many pathological conditions [17]. It is speculated that excessive ROS production in VS-ASCs shifts metabolism away from oxidative phosphorylation and toward glycolytic activity.

**Fig. 3:**
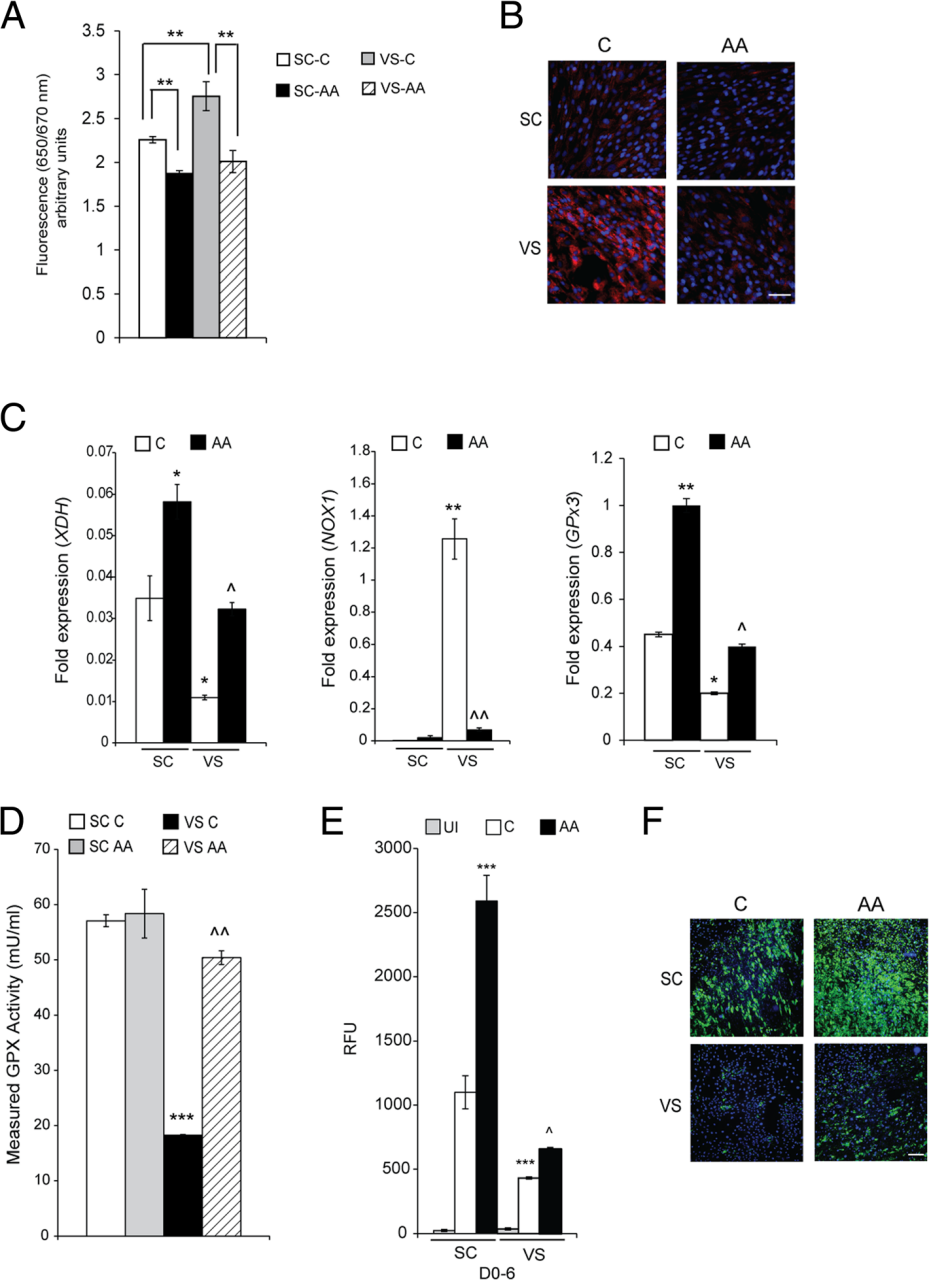
Ascorbic acid treatment improves ROS and adipogenic capacity of VS-ASCs. **a** Graph showing ROS levels of SC- and VS-ASCs each in triplicates from S17 when treated with ascorbic acid (AA—50 μM) for 48 h and then stained with CellROX™ Deep Red Reagent. Statistical significance was calculated by ANOVA. **p < 0.01. Similar results were obtained with another subject S14. **b** Representative images (× 40) showing fluorescence staining of ROS in S17 SC- and VS-ASCs when treated with AA for 48 h and then stained with CellROX™. Scale bar represents 100 μm. **c** Graphs showing fold expression of oxidative stress-related genes *XDH*, *NOX1*, and *GPx3* each in triplicates in SC- and VS-ASCs with or without AA from S17. Statistical significance was calculated by ANOVA. *p < 0.05 and **p < 0.01 when compared to SC-C; ^*p* < 0.05 and ^^*p* < 0.01 when compared to VS-C. **d** Graph showing GPx activity as measured by enzyme assay in SC- and VS-ASCs treated with AA each in triplicates from S17. Statistical significance was calculated by ANOVA. ***p < 0.001 when compared to SC C; ^^*p* < 0.01 when compared to VS C. **e** Graph showing relative AdipoRed staining levels of lipid droplets each in triplicates in S17 SC- and VS-ASCs that were treated with and without AA for the first 6 days (D0–6) during adipogenic stimulation. Statistical significance was calculated by ANOVA. ***p < 0.001 when compared to SC-C. ^*p* < 0.05 when compared to VS-C. **f** Representative images (× 10) showing AdipoRed staining of lipid droplets in S17 SC- and VS-ASCs with and without AA treatment. Scale bar represents 100 μm

Next, we investigated the effects of ascorbic acid (vitamin C), a water-soluble natural antioxidant and powerful scavenger of ROS [18, 19]. Ascorbic acid was found to potently reduce ROS levels of both SC- and VS-ASCs, as revealed by ROS-specific signal changes (Fig. 3a, b). Expression levels of antioxidant genes *XDH* and *GPx3* are upregulated by ascorbic acid in both ASCs, while pro-oxidant *NOX1* gene is significantly downregulated by ascorbic acid treatment in VS-ASCs (Fig. 3c). The activity of glutathione peroxidase (GPx), an antioxidant enzyme, is lower in VS-ASCs than in SC-ASCs, but significantly reversed by treatment with ascorbic acid (Fig. 3d). The abilities of both ASCs to differentiate into mature adipocytes are significantly improved when ascorbic acid is supplemented during adipogenic stimulation (Fig. 3e, f). In order to validate if the effects on adipogenesis are specifically mediated by ROS, SC-ASCs were incubated with ROS-causing hydrogen peroxide (H_2_O_2_) and treated with vitamin E, which is another antioxidant and specific ROS scavenger. As expected, H_2_O_2_ induced ROS and inhibited adipocyte differentiation of ASCs (Additional file 1: Figure S3). Treatment with vitamin E, however, at least partially reversed the ROS level and significantly improved the adipogenic defect induced by H_2_O_2_ (Additional file 1: Figure S3).

**Fig. 4:**
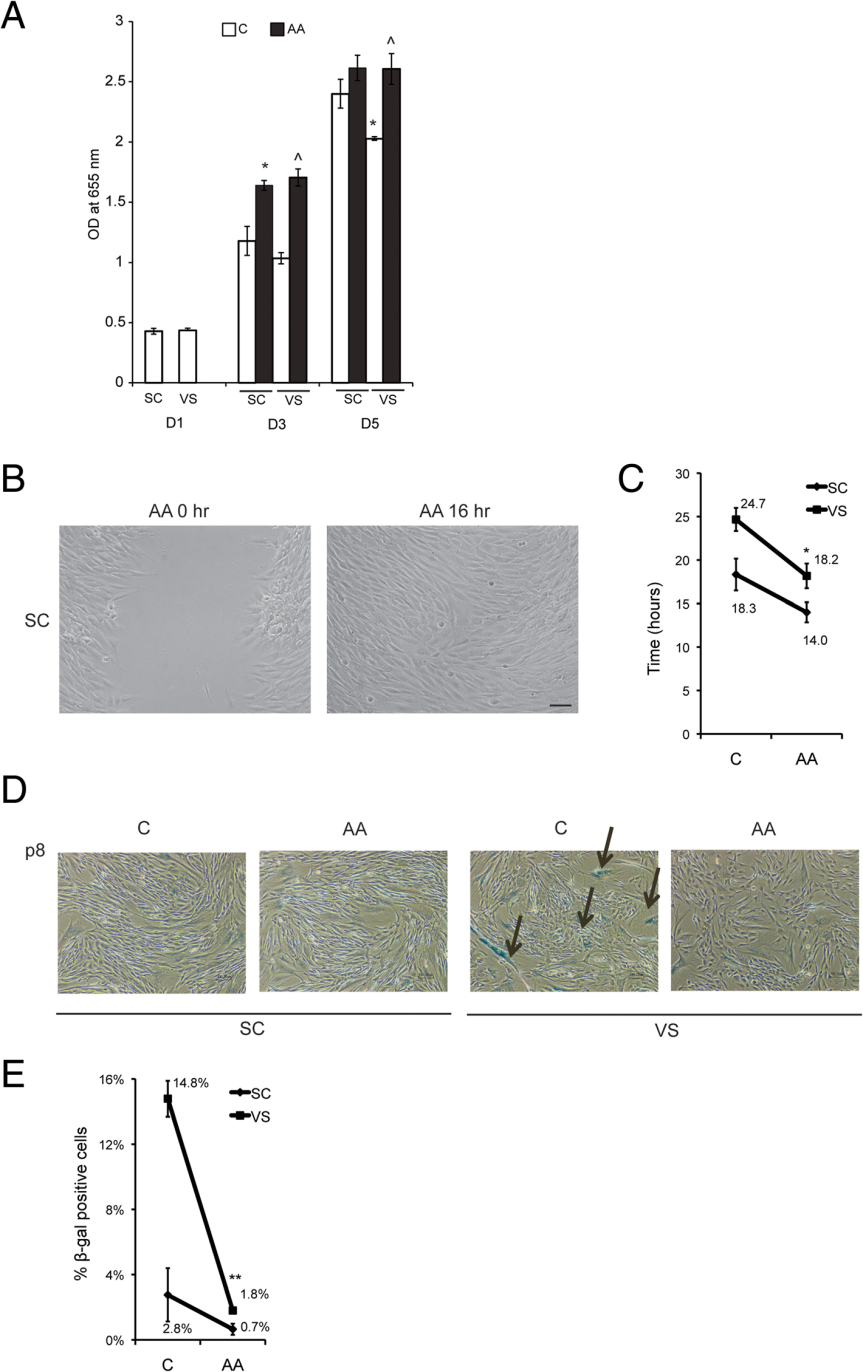
Ascorbic acid improves stem cell dysfunctions of VS-ASCs. **a** Proliferation assay using a methylene blue staining method each in triplicates in S17 SC- and VS-ASCs with and without 50 μM ascorbic acid (AA) treatment at various time points as indicated. Statistical significance was calculated by ANOVA. *p < 0.05 when compared to SC-C; ^*p* < 0.05 when compared to VS-C. **b** Representative images (× 10) showing migration assay by the scratch test for S17 SC-ASCs 16 h post AA treatment. Scale bar represents 100 μm. **c** Graph showing average hours taken for migration from S8, S14, and S17 SC- and VS-ASCs with and without AA treatment in the scratch test (*n* = 3). *p < 0.05 when compared to VS-C. **d** Representative images (× 10) showing senescence test using β-galactosidase in S8 SC- and VS-ASCs with and without AA treatment. **e** Quantification of the average percentage of cells exhibiting positive β-galactosidase staining from S18 and S19 SC- and VS-ASCs with and without AA treatment (*n* = 2). **p < 0.01 when compared to VS-C

In order to test if ascorbic acid also influences stem cell functions of ASCs, we measured cell proliferation rates by using methylene blue staining. The result indicates that VS-ASCs show slower proliferation, which can be substantially reversed by ascorbic acid supplementation (Fig. 4a). Similar to MSCs, ASCs exhibit migratory properties, in which VS-ASCs are more defective than SC-ASCs. Ascorbic acid treatment results in a significant shortening of migration time to fill scratched wounds in both SC- and VS-ASCs (Fig. 4b, c). Lastly, under standard culture condition, VS-ASCs start to exhibit senescence by passage 8 as revealed by beta-galactosidase staining, but the presence of ascorbic acid in culture media leads to significantly delayed senescence of VS-ASCs (Fig. 4d, e). Rather unexpectedly, Seahorse analysis indicates that treatment of ascorbic acid generally decreases both ECAR (glycolysis) and OCR (oxidative metabolism) activities in VS-ASCs (Additional file 1: Figure S4).

Collectively, our data indicate that antioxidants improve cellular dysfunctions of undifferentiated and differentiated ASCs caused by high oxidative stress. It is intriguing to speculate that inflammatory milieu of VS fat may render higher ROS of the stem cell population. This may in turn lead to defects of differentiation processes into mature adipocytes and account for deficient functions of VS adipocytes. Improvement of ROS with antioxidants may be a valid therapeutic approach in reversing pathological complications of VS fat. On the other hand, our results also point to the potential use of antioxidants for improving general ASC properties. Since ASCs, along with other MSCs, have been actively investigated for cell therapies, it is worth considering that inclusion of stable derivatives of ascorbic acid or other antioxidants in culture media may help achieve greater therapeutic abilities of ASCs for clinical applications.

## Conclusion

Our studies demonstrate that high ROS is observed in visceral fat-derived ASCs and associated with their altered oxidative metabolism and cellular dysfunctions, compared to subcutaneous fat-derived ASCs. Treatment with antioxidant ascorbic acid leads to improvement of adipocyte differentiation, proliferation, migration, and early senescence. These results point to depot-specific differences in oxidative stress that may be caused by molecular factors (e.g., pro-oxidant and antioxidant genes) and environmental cues (e.g., inflammation). In addition, our data suggest the potential use of antioxidants for improving ROS-mediated dysfunction of ASCs for future clinical applications.

## Methods

### Isolation and culture of ASCs

White adipose tissue (WAT) was isolated from the subcutaneous (abdominal region) depot and visceral depot (omental region) from human subjects undergoing bariatric surgery, which was approved by the Domain Specific Review Board at National Healthcare Group, Singapore. ASCs were isolated and enriched by serial passage culture of stromal vascular fractions (SVF) using ASC media (DMEM, 15% FBS, 1× GlutaMAX, 1× NEAA, Pen/Strep, 5 ng/ml basic FGF), as described previously [8, 10, 20]. Subject information is found in Additional file 1: Table S1. As previously described, we routinely checked the expression of MSC markers (CD73, CD90, and CD105) and trilineage differentiation in ASC culture. Only cells with similar passage numbers (up to p8) were used for any comparative studies. A stock solution of l-ascorbic acid (Sigma: A4403) was made freshly and added to media with a maximum of 48 h for ascorbic acid treatment.

### Cellular analyses

Adipogenesis of ASCs was performed as previously described with minor modifications [8, 10]. Briefly, after 2 days of overconfluence, cells were subjected to differentiation cocktail (0.5 mM isobutylmethylxanthine, 1 μM dexamethasone, 167 nM insulin, and 100 μM indomethacin) for 6 days, followed by maintenance media containing 167 nM insulin for 6 more days. Cells were stained with AdipoRed (Lonza). ROS was detected by a specific dye CellROX Deep Red Reagent (Thermo Fisher Scientific) and counter-stained with nuclear staining with Hoechst 33342 (Thermo Fisher Scientific), according to the manufacturer’s instructions. Stained cells were imaged by either ImageXpress Micro (Molecular Devices) or TS100 microscope (Nikon). Proliferation rates were measured by using the Methylene Blue and SpectraMax spectrophotometer (Molecular Devices) according to the previous publication [21]. Migration was measured by the scratch assay, recording time taken to fill the gap scratched with p200 pipet tips, as previously reported [22]. Senescence of cells was assessed by staining with beta-galactosidase (Thermo Fisher Scientific) as in the manufacturer’s instruction. For cellular and mitochondrial respiration analysis, Seahorse XF24 Analyzer (Agilent) was used accordingly to the manufacturer’s protocol. Six thousand ASCs were seeded in 24-well microplates and adhered for 1 day with ASC media. For the ascorbic acid treatment study, the drug (50 μM) was added at this stage. Then, cells were switched to the assay media (Seahorse XF Base Medium supplemented with 1% FBS, 1 mM pyruvate, 2 mM glutamine, and 10 mM glucose). OCR and ECAR were measured, with sequential addition of metabolic disruptors, oligomycin, carbonyl cyanide p-trifluoromethoxy-phenylhydrazone (FCCP), rotenone, and antimycin A. The data were normalized to the protein concentrations that had been estimated with the BCA protein assay, and analyzed using Prism version 5 software (GraphPad). For all the cellular assays, “triplicates” refers to technical replicates where experiments were performed in each cell type from three independent culture wells. *n* = 2 or 3 indicates that values were calculated from cells of two or three subjects.

### Quantitative real-time PCR

Real-time qPCR was performed as described previously [8, 10]. Briefly, total RNA was extracted using Trizol reagent (Thermo Fisher Scientific) and treated with DNase I to remove genomic DNA. cDNA conversion was done by the RevertAid H minus first-strand cDNA synthesis kit (Fermentas). Relative mRNA levels were calculated and normalized to that of GAPDH. The sequence of primers is found in Additional file 1: Table S2.

## Additional file

Additional file 1: **Figure S1.** Gene expression studies of additional ROS-related genes that are not included in Figure 1b. **Figure S2.** VS-ASCs have increased ROS when compared to SC-ASCs in the lower passage. **Figure S3.** Effects on adipogenesis are specifically mediated by ROS. **Figure S4.** Ascorbic acid treatment generally decreases both glycolytic and oxidative respirations. **Table S1.** List of subjects used for this study. **Table S2.** List of RT-qPCR primers-oligos 5′ to 3′. (DOCX 3731 kb)
