# Supplementary material for: Oxidative stress mediates depot-specific functional differences of human adipose-derived stem cells
Source: Stem Cell Res Ther. 2019 May 21;10:141. doi: 10.1186/s13287-019-1240-y (PMC6528291; doi:10.1186/s13287-019-1240-y)
Supplement: Supplementary file 1 — Figure S1. Gene expression studies of additional ROS-related genes that are not included in Figure 1b. Figure S2. VS-ASCs have increased ROS when compared to SC-ASCs in the lower passage. Figure S3. Effects on adipogenesis are specifically mediated by ROS. Figure S4. Ascorbic acid treatment generally decreases both glycolytic and oxidative respirations. Table S1. List of subjects used for this study. Table S2. List of RT-qPCR primers-oligos 5′ to 3′. (DOCX 3731 kb) [file 13287_2019_1240_MOESM1_ESM.docx]

**Additional file 1**

**Oxidative Stress Mediates Depot-specific Functional Differences of Human Adipose-derived Stem Cells**

Sandhya Sriram, Chengxiang Yuan, Smarajit Chakraborty, Winson Tay, Min Park, Asim Shabbir, Sue-Anne Toh, Weiping Han, Shigeki Sugii

**Supplementary Methods**

**Image analysis**

The images were collected using an Olympus IX71 Inverted Microscope (Applied Precision DeltaVision Deconvolution microscope system) equipped with 20X, objective lens with a mercury arc bulb (OLYMPUS U-RFL-T: Mercury 150W) as the illumination source. Image capture was performed with the CoolSnap HQ, a fast, high resolution, high quantum efficiency, cooled CCD camera (Photometrics CoolSNAP HQ2 (CCD).

**Treatment with hydrogen peroxide and vitamin E**

ASCs were treated with 50μM hydrogen peroxide with or without 50μM α-tocopherol as vitamin E (Sigma) for two days before ROS detection described in the Methods section. Adipogenesis assay was conducted as described in the Methods section. 50μM hydrogen peroxide with or without 50μM α-tocopherol as vitamin E was included from Day -2 to Day 0 (before adipogenesis was initiated for 12 days).


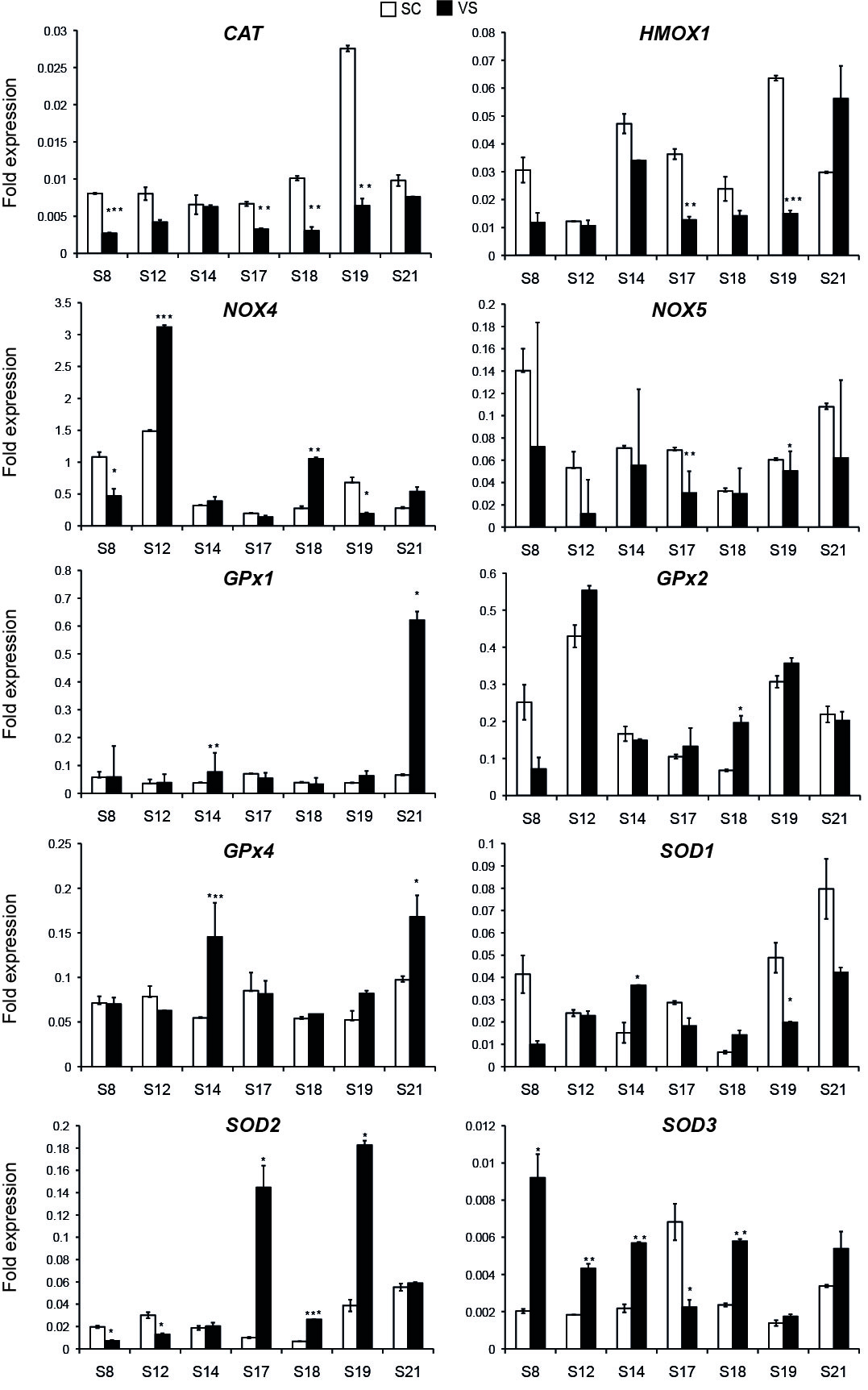


**Figure S1**

**Gene expression studies of additional ROS-related genes that are not included in Figure 1B**

The same analysis as in Figure 1B. *p<0.05, **p<0.01, ***p<0.001 when compared to SC.


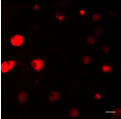

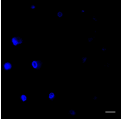

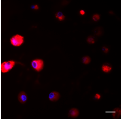

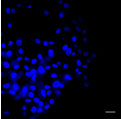

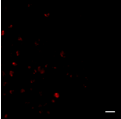

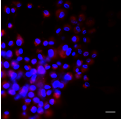

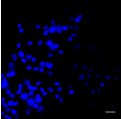

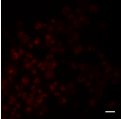

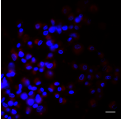

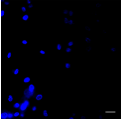

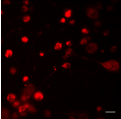

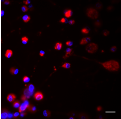


ROS Nuclei Merge ROS Nuclei Merge

SC

VS

S9 S22

**A**

Fluorescence 650/670 nm

(AU)

**B**

*

*

**Figure S2**

**VS-ASCs have increased ROS when compared to SC-ASCs in lower passage**

(A) Representative images (20X) showing fluorescence staining of ROS (red) and nuclei (blue) and merged in S9 and S22 SC- and VS-ASCs of lower passage (p3) when stained with CellROX™ and Hoechst 33342, respectively. All the images obtained and displayed are in a similar setup. Scale bar represents 100μm. (B) Graphs showing fluorescence intensity of SC- and VS-ASCs stained with CellROX™ Deep Red Reagent normalized to the nuclei intensities performed in triplicates. Statistical significance was calculated by ANOVA. *p<0.05 when compared to SC.


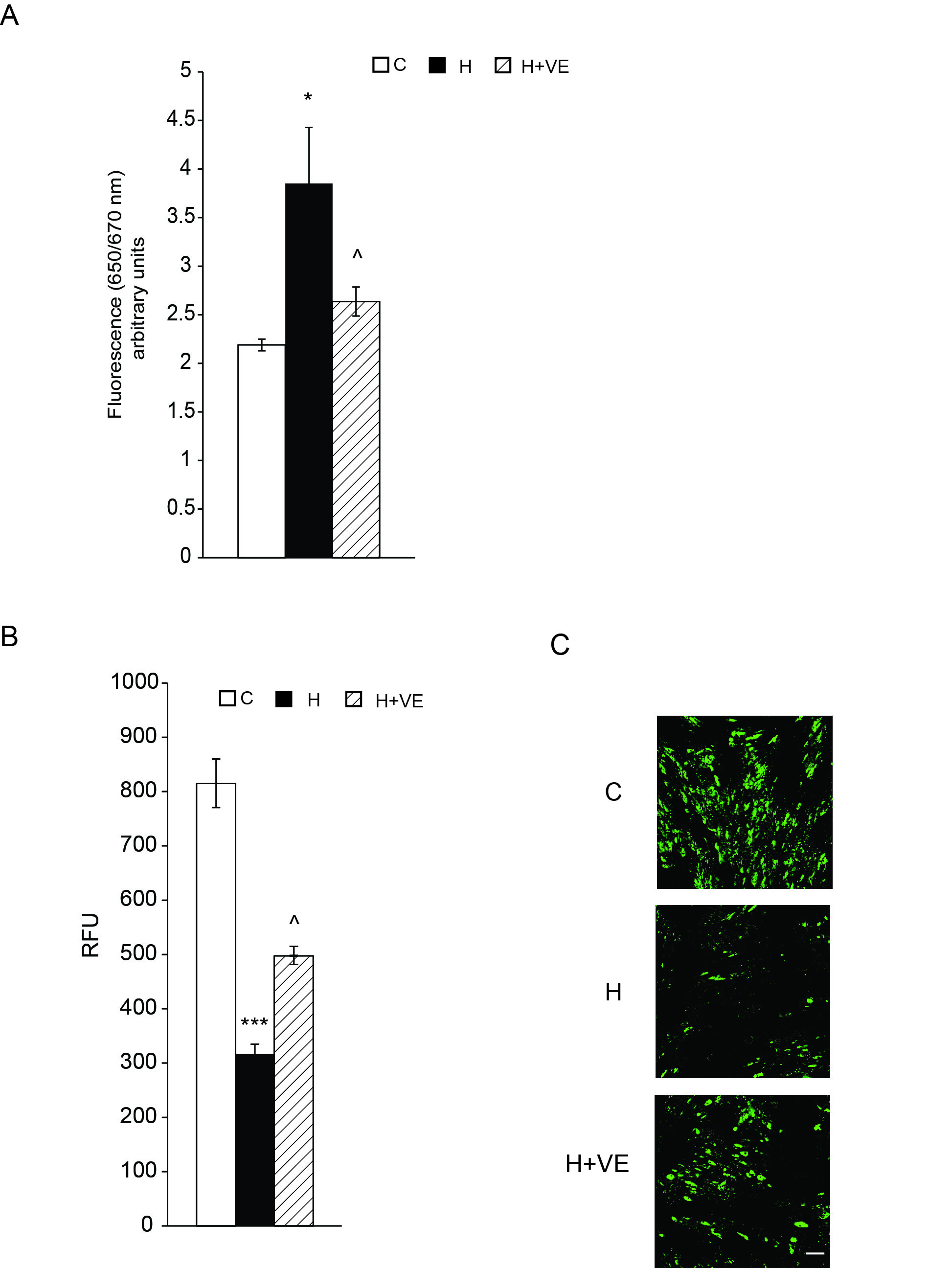


**Figure S3**

**Effects on adipogenesis are specifically mediated by ROS**

# (A) Graph showing ROS levels of SC-ASCs in triplicates from S17 when treated with hydrogen peroxide (H) with or without vitamin E (VE) for 48 hours, and then stained with CellROX™ Deep Red Reagent. Statistical significance was calculated by ANOVA. *p<0.05 when compared to C; ^p<0.05 when compared to H. (B) Graph showing relative AdipoRed staining levels of lipid droplets in triplicates in S17 SC-ASCs that were treated with hydrogen peroxide and with or without vitamin E for 48 hours during adipogenic stimulation. Statistical significance was calculated by ANOVA. ***p<0.001 when compared to C. ^p<0.05 when compared to cells treated with H. (C) Representative images (10X) showing AdipoRed staining of lipid droplets from the same cell sample groups as in (B). Scale bar represents 100µm.

B

A


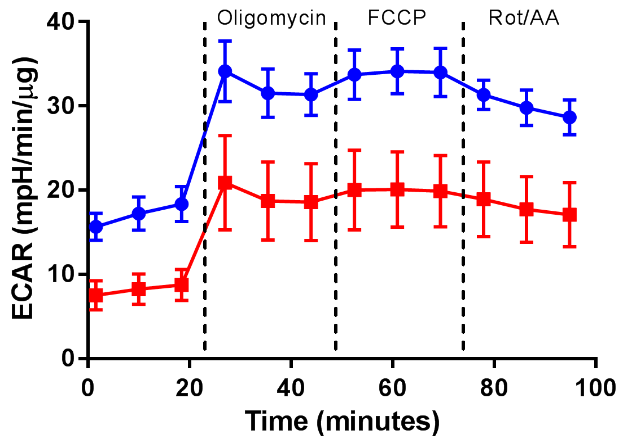


**

**

**

**

**AA**

**C**

**S14**


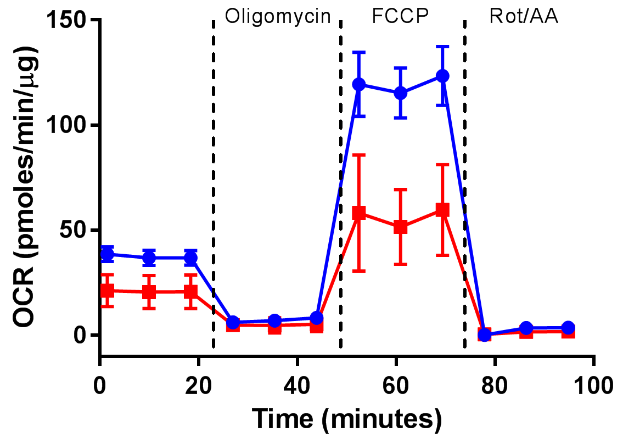


*

**

**
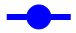

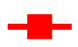
**

**
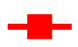

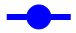

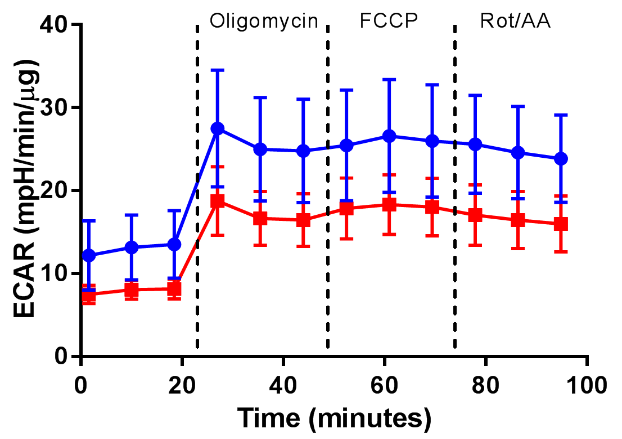

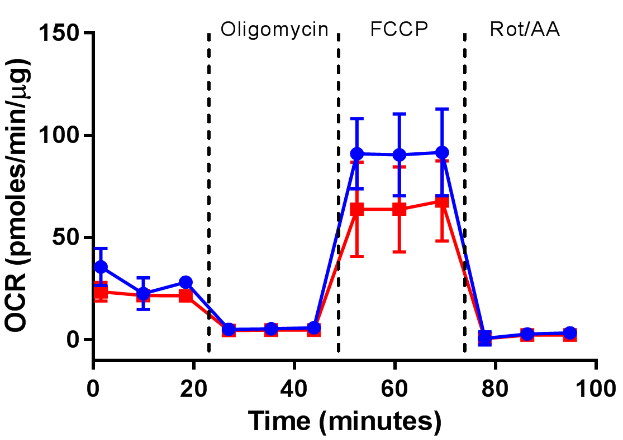
**

**S17**

D

C

**AA**

**C**

**Figure S4**

**Ascorbic acid treatment generally decreases both glycolytic and oxidative respirations**

Graphs showing (A, C) ECAR (extracellular acidification rate) and (B, D) OCR (oxygen consumption rate) for VS-ASCs in presence (AA) or absence (C) of ascorbic acid analyzed by Seahorse XF each in triplicates from S14 and S17. Statistical significance was calculated by ANOVA. *p<0.05, **p<0.01.

**Supplementary Table S1**

List of subjects used for this study. T2DM, type 2 diabetes; NDM, non-diabetic

| **Subject** | **Age** | **Sex** | **Race** | **Height**  **(cm)** | **Weight**  **(kg)** | **BMI** | **T2DM/ NDM** |
| --- | --- | --- | --- | --- | --- | --- | --- |
| S8 | 39 | F | Chinese | 157 | 98 | 40 | T2DM |
| S9 | 26 | F | Malay | 173 | 113 | 38 | T2DM |
| S12 | 25 | F | Chinese | 160 | 98 | 38 | NDM |
| S14 | 25 | F | Malay | 167 | 150 | 54 | T2DM |
| S17 | 34 | F | Chinese | 157 | 134 | 54 | NDM |
| S18 | 33 | F | Chinese | 165 | 101 | 37 | T2DM |
| S19 | 34 | F | Chinese | 159 | 89 | 35 | NDM |
| S21 | 21 | F | Malay | 165 | 105 | 39 | NDM |
| S22 | 46 | M | Malay | 172 | 119 | 40 | NDM |

**Supplementary Table S2**

List of RT-qPCR primers-Oligos 5’ to 3’

| **Gene** | **Forward** | **Reverse** |
| --- | --- | --- |
| *NOX1* | ATTCCAGTGTGCAGACCACA | CGGTGAGGAAGAGACGGTAG |
| *NOX2* | CCTGAAGGGTTCTGCACATT | GCTGGAGAAGACCACTTTGG |
| *NOX3* | TCACAAACTGGTCGCCTATG | CAGGGTTGAGGTAGCTCTCG |
| *NOX4* | GCCGAACACTCTTGGCTTAC | GTTGAGGGCATTCACCAGAT |
| *NOX5* | CCTCATGTTCATCTGCTCCA | AACAAGATTCCAGGCACCAG |
| *GPx1* | TTGACATCGAGCCTGACATC | CAAGGTGTTCCTCCCTCGTA |
| *GPx2* | AGATGTGGCCTGGAACTTTG | TAAGGCTCCTCAGGACTGGA |
| *GPx3* | ATGCTGGCAAATACGTCCTC | CTCCTGGTTCCTGTTTTCCA |
| *GPx4* | GATGAAGATCCAACCCAAGG | ATAGTGGGGCAGGTCCTTCT |
| *XDH* | GCATATCATTGGTGCTGTGG | GGTCCCCTTTCTCGATCTTC |
| *NOS3* | TGTCTGCATGGACCTGGATA | CTGGCCTTCTGCTCATTCTC |
| *HMOX1* | TCCGATGGGTCCTTACACTC | TAAGGAAGCCAGCCAAGAGA |
| *CAT* | CTGGAGAAGTGCGGAGATTC | AGTCAGGGTGGACCTCAGTG |
| *SOD1* | GAAGGTGTGGGGAAGCATTA | ACATTGCCCAAGTCTCCAAC |
| *SOD2* | TTGGCCAAGGGAGATGTTAC | AGTCACGTTTGATGGCTTCC |
| *SOD3* | CAACAGACACCCTCCACTCT | TCGGTACAAATGGAGGCCTT |
| *GAPDH* | caaggtcatccatgacaactttg | ggccatccacagtcttctgg |
